# Supplementary material for: High-performance fault-tolerant quantum computing with many-hypercube codes
Source: Sci Adv. 2024 Sep 4;10(36):eadp6388. doi: 10.1126/sciadv.adp6388 (PMC11373590; doi:10.1126/sciadv.adp6388)
Supplement: Supplementary file 1 — Supplementary Text Figs. S1 to S6 Tables S1 to S3 [file sciadv.adp6388_sm.pdf]

Science Advances
AAAS

Supplementary Materials for

High-performance fault-tolerant quantum computing with many-hypercube codes

Hayato Goto

Corresponding author: Hayato Goto, hayato.goto@riken.jp

Sci. Adv. **10**, eadp6388 (2024)
DOI: 10.1126/sciadv.adp6388

This PDF file includes:

Supplementary Text
Figs. S1 to S6
Tables S1 to S3

Supplementary Text

Results at level 2 using the Steane method for error detection

The crosses in Fig. S1 show the results at level 2 using the Steane method in error-detection gadgets instead of the flag-based method in the main text (see Table S3 for the numbers of trials of the simulation). As found from Fig. S1, the Steane method leads to no performance improvement and a larger space overhead, compared to the flag-based method. This is the reason why we use the flag-based method at level 2.

Results at level 4 using the encoder in Fig. 5B

The squares and triangles in Fig. S2 show the results at level 4 using the encoder in Fig. 5B, instead of Fig. 5G, with $L_{\mathrm{D}} = 1$ and 2 (see Materials and Methods), respectively (see Table S3 for the numbers of trials of the simulation). As found from Fig. S2, the encoder in Fig. 5B with $L_{\mathrm{D}} = 1$ results in the rapid increase of the space overhead with respect to p_{circ} and also the one with $L_{\mathrm{D}} = 2$ leads to a lower overhead but higher logical controlled-NOT (CNOT) error probabilities, compared to the encoder with Fig. 5G. This is the reason why we propose the encoder in Fig. 5G at level 4.

Details and flowcharts of the proposed level-by-level minimum distance decoding

Here we explain our proposed decoding method in more detail using flowcharts. As an example, we focus on the level-3 case.

Figure S3 shows the whole process of the decoding in the level-3 case. Since the subroutines Sub1 and Sub2 are well explained in Materials and Methods, here we explain the subroutine Sub3 in detail.

Figure S4 shows the process of Sub3. The subroutine Sub31 for b_2=6 is shown in Fig. S5, where N_1 to N_5 denote the numbers of the minimum-distance candidates of the first to fifth level-2 blocks, respectively, selected in Sub2. If the total number of candidates, $N_1N_2N_3N_4N_5$, is larger than a preset threshold N_{th3}, we reduce the number by randomly choosing one of the candidates from the level-2 block with the largest N, in order to keep the decoding time short. In this work, we set N_{th3} to 10^5. Then we determine the encoded bit string of the sixth level-2 block using the n_1-th to n_5-th minimum-distance candidates of the first to fifth level-2 blocks, respectively, according to the parity-check condition (Z-stabilizer condition). More concretely, the encoded bit string of the sixth level-2 block is determined as follows:

$$x^{(2)}_{i\prime,j\prime,6} = x^{(2)}_{i',j',1} + x^{(2)}_{i',j',2} + x^{(2)}_{i',j',3} + x^{(2)}_{i',j',4} + x^{(2)}_{i',j',5} \text{ (mod 2 and } i', j' = 1, \dots, 4)$$

In general, the level-2 encoded bit string determined above is not included in the minimum-distance candidates of this block selected in Sub2. Therefore, in the subroutine Sub32, we evaluate the distance of this encoded bit string. Figure S6 shows Sub32 for b_2=6, where M_1 to M_6 denote the numbers of the minimum-distance candidates of the first to sixth level-1 blocks, respectively, selected in Sub1 in the sixth level-2 block. If M_1+M_2+M_3+M_4+M_5+M_6 is larger than a preset threshold M_{th2}, we reduce the number by randomly choosing one of the candidates from the level-1 block with the largest M. In this work, we set M_{th2} to 6. We first determine the encoded bit strings of the five level-1 blocks other than the b-th level-1 block in the sixth level-2 block using the m_b-th minimum-distance candidate of the b-th level-1 block selected in Sub1 and the level-2 encoding bit string determined in Sub31 according to the parity-check condition and

the definition of the encoded Z operator. More concretely, when b=1, the encoded bit strings of the five level-1 blocks are determined as follows:

$$x_{i',2,6}^{(1)} = x_{i',1,6}^{(1)} + x_{i',1,6}^{(2)} \text{ (mod 2 and } i' = 1, \ldots, 4)$$

$$x_{i',3,6}^{(1)} = x_{i',2,6}^{(1)} + x_{i',2,6}^{(2)} \text{ (mod 2 and } i' = 1, \ldots, 4)$$

$$x_{i',5,6}^{(1)} = x_{i',2,6}^{(1)} + x_{i',1,6}^{(2)} + x_{i',2,6}^{(2)} + x_{i',3,6}^{(2)} + x_{i',4,6}^{(2)} \text{ (mod 2, and } i' = 1, \ldots, 4)$$

$$x_{i',4,6}^{(1)} = x_{i',5,6}^{(1)} + x_{i',3,6}^{(2)} \text{ (mod 2 and } i' = 1, \ldots, 4)$$

$$x_{i',6,6}^{(1)} = x_{i',5,6}^{(1)} + x_{i',4,6}^{(2)} \text{ (mod 2 and } i' = 1, \ldots, 4)$$

In general, the level-1 encoded bit strings determined above are not included in the minimum-distance candidates of the level-1 blocks selected in Sub1. Therefore, we evaluate the distances of the encoded bit strings. This distance evaluation can easily be achieved at level 1. By summing the distances of the six level-1 blocks, we obtain the distance of the sixth level-2 block. In Sub32, we finally select the minimum distance of the sixth level-2 block among all the choices of a level-1 block and its minimum-distance candidates.

As shown in Fig. S5, then we evaluate the distances of the level-3 codewords by summing the distances of their six level-2 blocks. We finally select level-3 encoded bit strings with minimum distance, as shown in Fig. S4.

The decoding of the level-4 many-hypercube code is done in a similar manner to the above level-3 case. In the level-4 case, we set the level-4 threshold N_{th4} corresponding to the above level-3 threshold N_{th3} to the same value 10^5 and the level-3 threshold M_{th3} corresponding to the above level-2 threshold M_{th2} to 12.

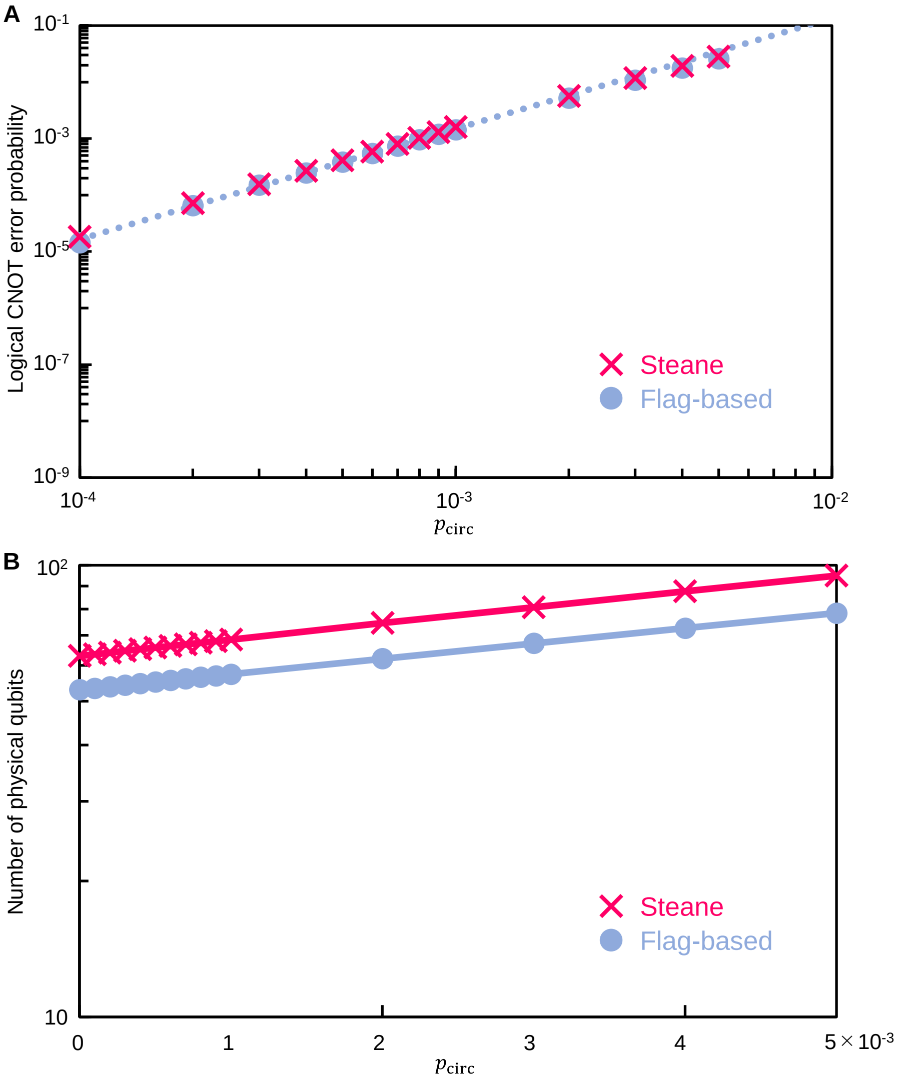

Fig. S1. Results at level 2 with the Steane method for error detection. (A) Logical controlled-NOT (CNOT) error probability. (B) The total number of physical qubits for the zero-state encoder.

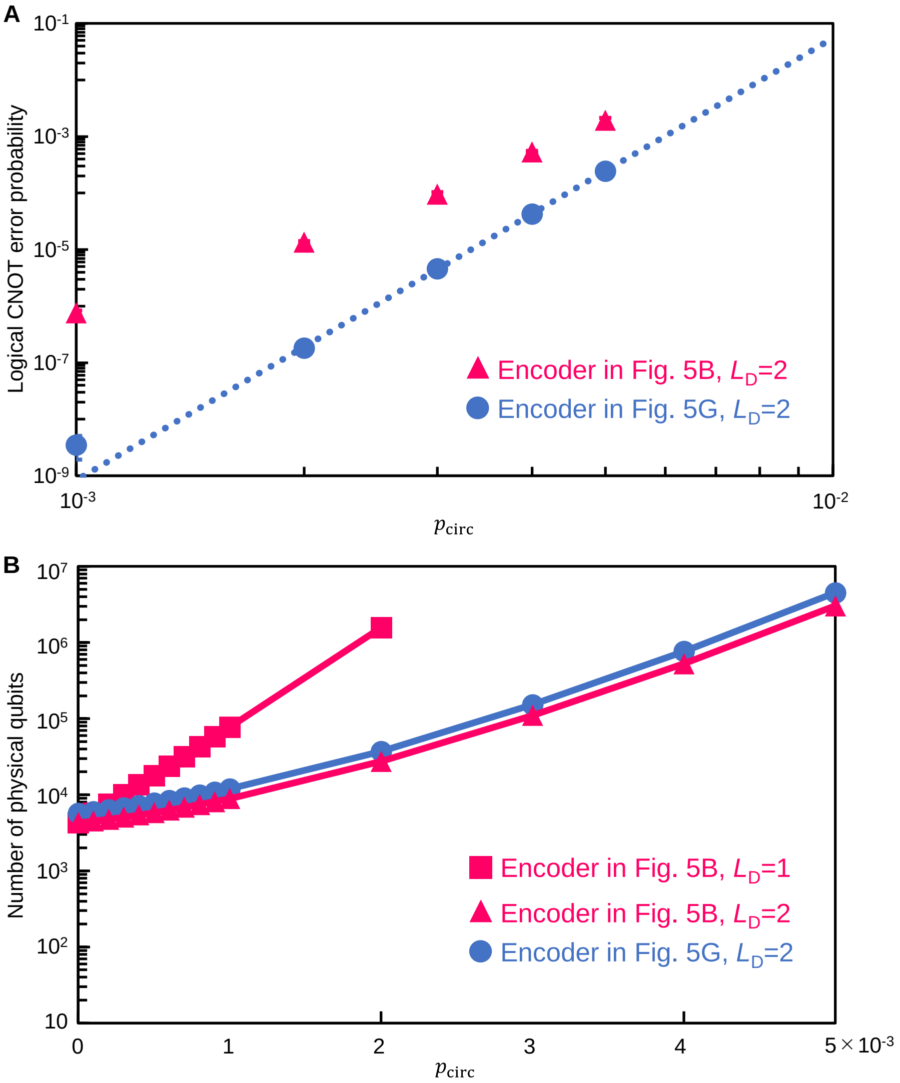

Fig. S2. Results at level 4 with the zero-state encoder in Fig. 5B. (A) Logical controlled-NOT (CNOT) error probability. (B) The total number of physical qubits for the zero-state encoder.

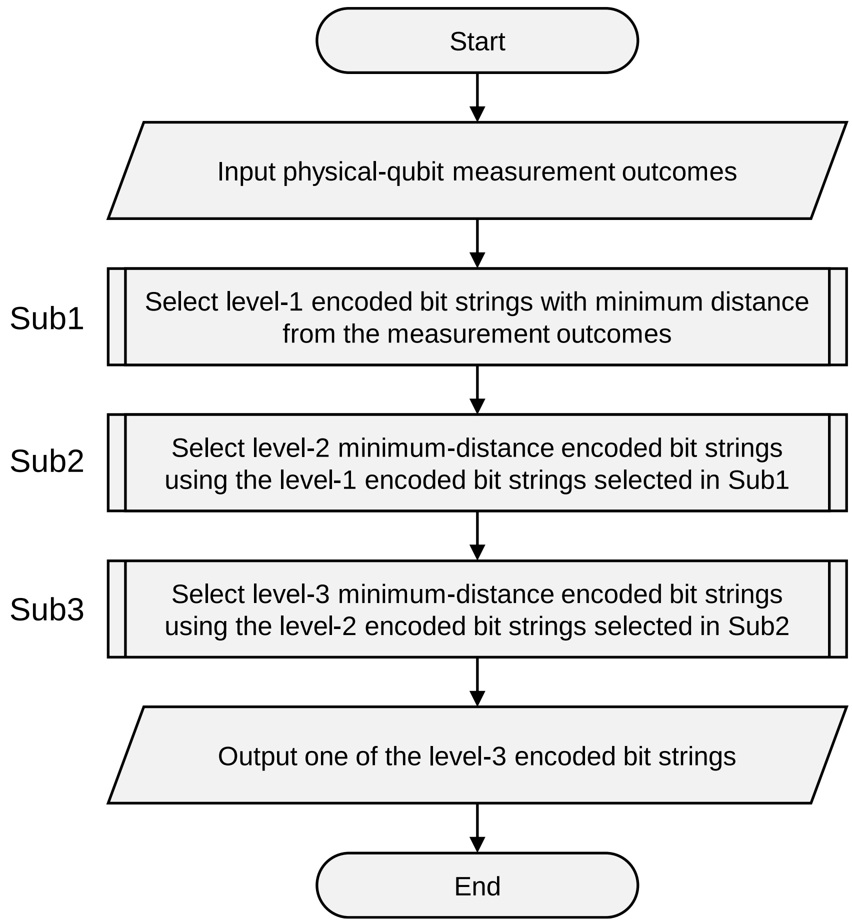

Fig. S3. Flowchart of the whole process of the level-by-level minimum distance decoding in the level-3 case. See Fig. S4 for the details of the subroutine Sub3.

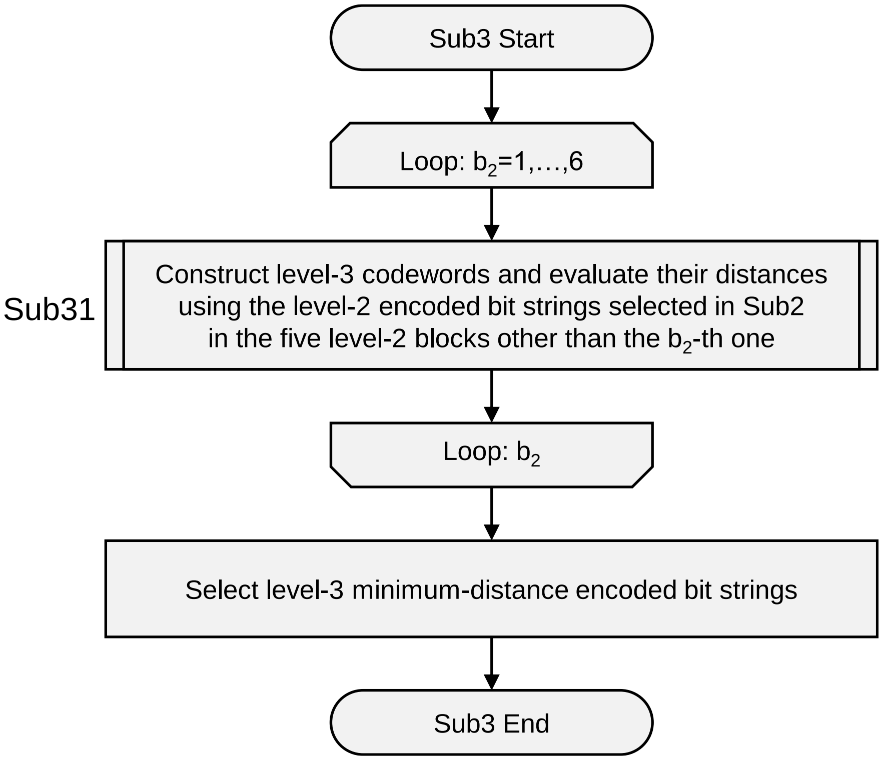

Fig. S4. Flowchart of the subroutine Sub3 in Fig. S3. See Fig. S5 for the subroutine Sub31 when b_2=6.

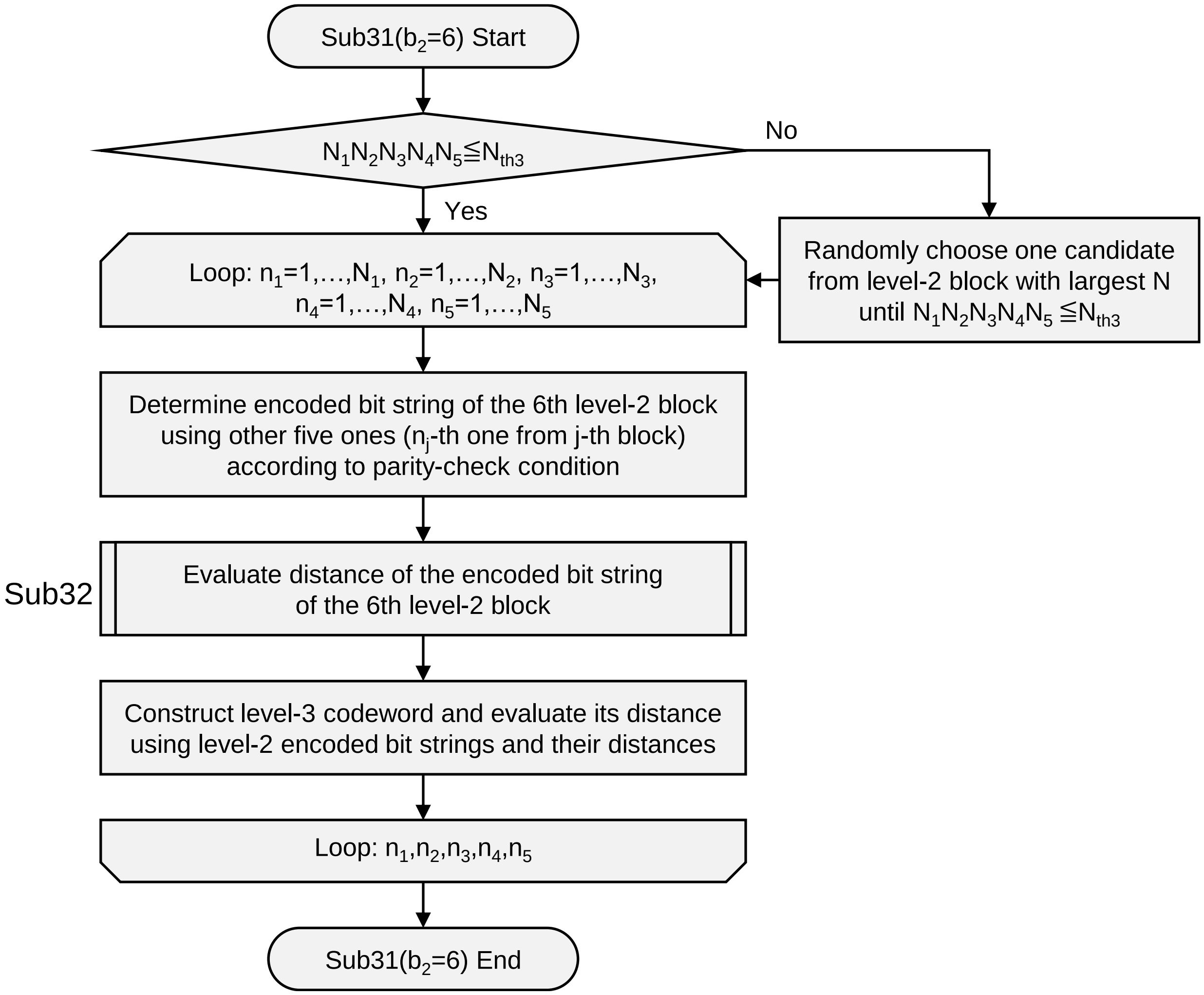

Fig. S5. Flowchart of the subroutine Sub31 in Fig. S4 when b_2=6. N_b denotes the number of candidates of the b-th level-2 block selected in Sub2. See Fig. S6 for the subroutine Sub32.

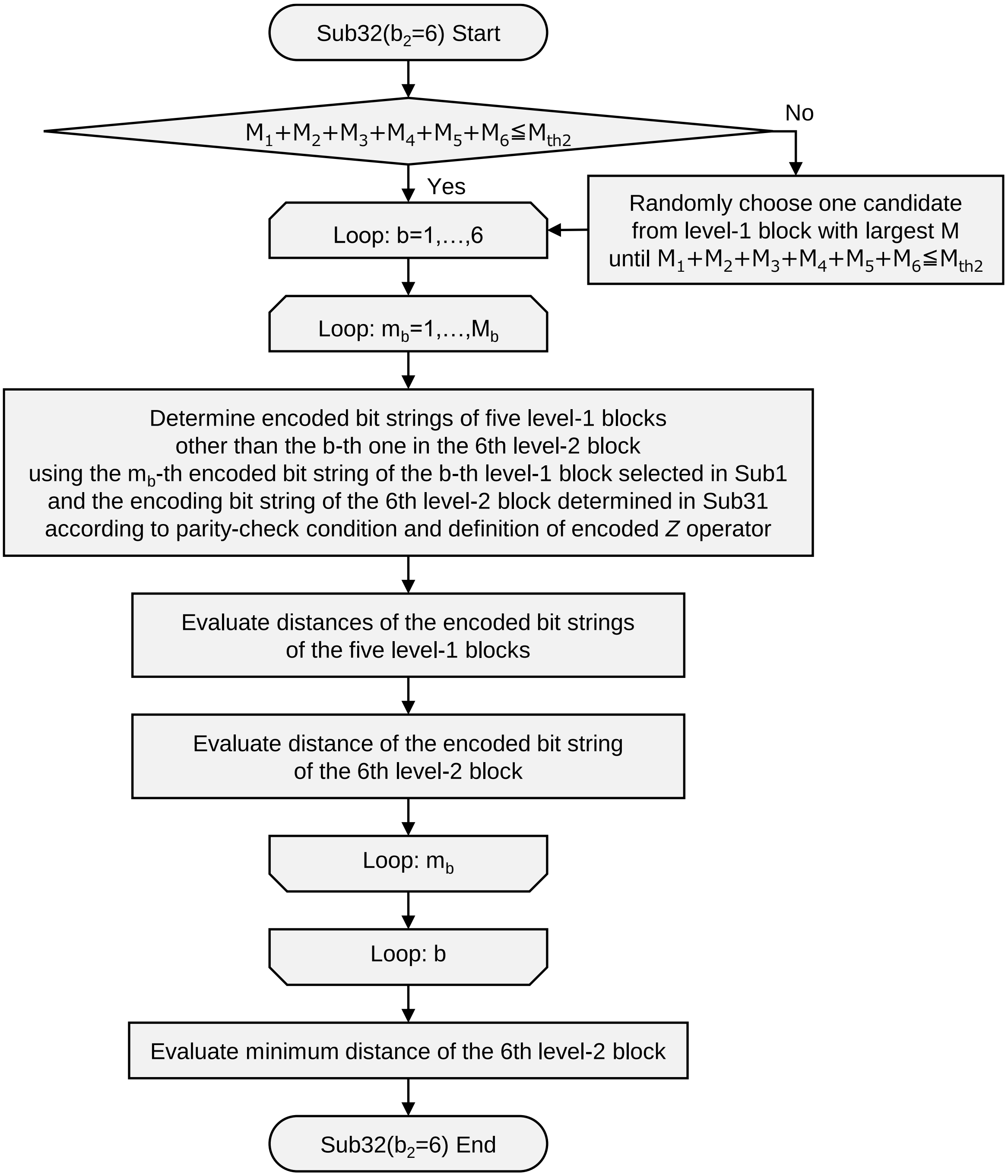

Fig. S6. Flowchart of the subroutine Sub32 in Fig. S5. M_b denotes the number of candidates of the b-th level-1 block selected in Sub1.

Table S1. The numbers of trials of the simulation for Fig. 4. The values in parentheses are those of p_{circ}.

Figure	Level 2	Level 3	Level 4
Fig. 4A	560000	560000	560000
Fig. 4B	5600000	5600000	5600000
Fig. 4C	20000000 (0.001–0.009)<br>1000000 (0.01–0.1)	20000000 (0.002–0.008)<br>1200000 (0.01–0.1)	20000000 (0.015–0.025)<br>200000 (0.03–0.05)<br>120000 (0.06–0.1)

Table S2. The numbers of trials of the simulation for Fig. 6. The values in parentheses are those of p_{circ}.

Level 2	Level 3	Level 4
56000	560000 (0.0001–0.0009)<br>56000 (0.001–0.005)	5600 (0.0001–0.0009)<br>560000 (0.001)<br>112000 (0.002)<br>11200 (0.003)<br>1120 (0.004)<br>560 (0.005)

Table S3. The numbers of trials of the simulation for Figs. S1 and S2. The values in parentheses are those of p_{circ}.

Crosses	Squares	Triangles
56000	5600 (0.0001–0.0009)<br>1120 (0.001–0.002)	560 (0.0001–0.0009,0.003–0.005)<br>56000 (0.001)<br>11200 (0.002)
